# The Necessary Peroxide of Hydrogen

**Published:** 1890-09

**Authors:** Robert T. Morris

**Affiliations:** New York


### The Necessary Peroxide of Hydrogen.

BY ROBERT T. MORRIS, M.D., OF NEW YORK.

Read in the Section of Surgery and Anatomy, at the Forty-first Annual Meeting of the American Medical Association, held at Nashville, Tenn., May, 1890.

Stop suppuration! That is the duty that is imposed upon us when we fail to prevent suppuration.

As the ferret hunts the rat, so does peroxide of hydrogen follow pus to its narrowest hiding place, and the pyogenic and other micro-organisms are as dead as the rat that the ferret catches, when the peroxide is through with them. Peroxide of hydrogen $H_2O_2$ in the strong 15-volume solution is almost as harmless as water, and yet, according to the testimony of Gifford, it kills anthrax spores in a few minutes.

For preventing suppuration we have bichloride of mercury, hydronapthol, carbolic acid, and many other antiseptics, but for stopping it abruptly and for sterilizing a suppurating wound we have only one antiseptic that is generally efficient so far as I know, and that is the strong peroxide of hydrogen. Therefore I have qualified it, not as "good," not as "useful," but as "necessary."

In abscess of the brain, where we could not thoroughly wash the pus out of tortuous canals without injurying the tissues, the $H_2O_2$ injected at a superficial point, will follow the pus and throw it out, too, in a foaming mixture. It is best to inject a small quantity, wait until foaming ceases, and repeat injections until the last one fails to bubble. Then we know that the pus cavity is chemically clean, as far as live microbes are concerned.

In appendicitis, we can open the abscess, inject peroxide of hydrogen, and so thoroughly sterilize the pus cavity that we need not fear infection of the general peritoneal cavity if we wish to separate intestinal adhesions and remove the appendix vermiformis. Many a patient, who is now dead, could have been saved if peroxide of hydrogen had been thus used when he had appendicitis.

The single means at our disposal allows us to open the most extensive psoas abscess without dread of septic infection following.

In some cases of purulent conjunctivitis we can build a little wall of wax about the eye, destroy all pus with peroxide of hydrogen and cut the suppuration short. Give the patient ether if the $H_2O_2$ causes too much smarting. It is only in the eye, in the nose and in the urethra that peroxide of hydrogen will need to be preceded by cocaine (or ether) for the purpose of quieting the smarting, for it is elsewhere almost as bland as water.

It is possible to open a large abscess of the breast, wash it out with $H_2O_2$ and have recovery ensue under one antiseptic dressing, without the formation of another drop of pus.

Where cellular tissues are breaking down and in old sinuses, we are obliged to make repeated applications of the $H_2O_2$ for many days and in such cases I usually follow it with balsam of Peru, for balsam of Peru, either in fluid form or used with sterilized oakum, is a most prompt encourager of granulation.

If we apply $H_2O_2$ on a probang to diphtheritic membranes at intervals of a few moments, they swell up like wipped cream and come away easily leaving a clean surface. The fluid can be snuffed up into the nose and will render a fetid ozæna odorless.

It is unnecessary for me to speak of further indications for its use, because wherever there is pus we should use peroxide of hydrogen. We are all familiar with the old law, "*Ubi pus, ibi evacua*," and I would change it to read, "*Ubi pus, ibi evacua, ibi hydrogenum peroxidum infunde.*" That is the rule. The exceptions which prove the rule are easily appreciated when we have them to deal with.

Peroxide of hydrogen is an unstable compound and becomes weaker as oxygen is given off, but Marchand's 15-volume solution will retain active germicidal powers for many months if kept tightly corked in a cold place. The price of this manufacturer's preparation is about 75 cents per lb., and it can be obtained from any large drug house in this country. When using the $H_2O_2$ it should not be allowed to come into contact with metals if we wish to preserve its strength, as oxygen is then given off too rapidly.

$H_2O_2$ must be used with caution about the hair if the color of the hair is a matter of importance to the patient, for this drug, under an alias, is the golden hair bleach of the *nymph's dispare*, and a dark-haired man with a canary-colored moustache is a stirring object.

